# Focal seizures during simulated driving: A pilot study

**DOI:** 10.1002/epi.70198

**Published:** 2026-03-13

**Authors:** Emma Nilsson, Mirta Zelenika Zeba, Joakim Strandberg, Peter Lundgren, Johan Zelano

**Affiliations:** ^1^ Volvo Cars Safety Center Volvo Cars Gothenburg Sweden; ^2^ Department of Clinical Neuroscience, Institute of Neuroscience and Physiology Sahlgrenska Academy, University of Gothenburg Gothenburg Sweden; ^3^ Department of Clinical Neurophysiology Sahlgrenska University Hospital Gothenburg Sweden; ^4^ Department of Molecular and Clinical Medicine, Institute of Medicine Sahlgrenska Academy, University of Gothenburg Gothenburg Sweden; ^5^ Prehospen–Center for Prehospital Research University of Borås Borås Sweden; ^6^ Department of Thoracic Surgery and Cardiology Sahlgrenska University Hospital Gothenburg Sweden; ^7^ Center for Digital Health Sahlgrenska University Hospital Gothenburg Sweden; ^8^ Department of Neurology Sahlgrenska University Hospital, member of European Reference Network EpiCARE Gothenburg Sweden; ^9^ Wallenberg Center for Molecular and Translational Medicine University of Gothenburg Gothenburg Sweden

**Keywords:** driver license, driving, epilepsyseizurevehicle

## Abstract

Cars are increasingly equipped with technology that can be used to analyze driver behavior and alertness, often referred to as driver monitoring systems (DMS). Although initially mainly used to track drivers' attention, DMS are now expanding in the hope of detecting additional forms of driver impairment that may jeopardize driving, such as drowsiness and those caused by sudden medical emergencies. To explore the potential for the latter, we conducted a pilot study to investigate whether technology in modern vehicles, such as eye tracking and driving behavior sensing, can detect abnormalities during seizures. We included 10 patients with focal epilepsy, all of whom had high seizure frequencies and a history of focal impaired awareness seizures. In three subjects, we recorded three definite and one possible electrographic seizure. All seizures were focal, with no motor features. We evaluated driving performance, saccade frequency, eye blink rate, and gaze direction. No clear impact of seizures on driving performance was observed, and eye‐related measures showed inconsistent changes. Future studies should explore additional indicators and assess the potential to detect more severe seizures that may have a greater impact on driving performance.

## 1 INTRODUCTION

Inability to drive is one of the major impacts of an epilepsy diagnosis, with psychosocial consequences for quality of life.^1^, ^2^ Driving can also hinder adequate epilepsy treatment if seizures are underreported or antiseizure medication (ASM) therapy revision declined for fear of losing driving privileges.^2^, ^3^, ^4^ Regulations allow many persons with epilepsy to drive,^5^ but risks of vehicle accidents are increased.^6^, ^7^ Crash and car simulator studies indicate that loss of consciousness in tonic–clonic seizures and focal impaired awareness seizures impair driving abilities significantly.^8^, ^9^, ^10^, ^11^ The literature is more heterogenous in the absence of clinical seizures.^12^ Cognitive disability and interictal discharges have been associated with impaired driving.^13^, ^14^

Medical conditions cause a substantial number of vehicle accidents and are of great interest to the car industry. Cars are increasingly equipped with technology that can be used to monitor driver behavior and alertness to support drivers and increase safety. Hopes have been raised that more technological support could allow persons with epilepsy to drive to a greater extent than today.^2^ Some seizure symptoms, like altered tone or convulsions should be detectable. It would also be advantageous if systems could also detect more subtle signs of focal seizures, for instance by additional interpretation of eye tracking or driving behavior. Studies in epilepsy have so far focused on the impact of seizures on driving ability.^8^, ^13^, ^14^ In this pilot study, we asked whether it is feasible to study technology that can detect subtle seizures and be made available in modern cars, such as eye tracking and driving behavior sensing. We wanted to assess the scale of data collection, explore whether eye tracking or driving sensors detect abnormalities during seizures, and gain insights for future studies.

## 2 MATERIALS AND METHODS

### 2.1 Study design and patients

This pilot study was a collaboration by Volvo Cars and Sahlgrenska University Hospital. Inclusion criteria were the following: age = 18+ years, informed consent, epilepsy, seizure frequency and type deemed suitable, and capability to operate the simulator. The exclusion criterion was any condition or history that would constitute an increased risk. Participants gave written consent. Before data collection, they practiced the simulator for 15 min.

### 2.2 Data collection

Following an interview regarding medical history, the experimental drive consisted of as many driving sessions as the patient wanted, with a total duration of no more than 3 h. Each session lasted 30–60 min, followed by an at least 10‐min break. After each session, participants answered questions about driving and seizure symptoms.

#### 2.2.1 Clinical characteristics

Age, sex, epilepsy type, semiology, seizure frequency, current ASMs, and imaging data were recorded in a pseudonymized form.

#### 2.2.2 Biosensors

Electroencephalography (EEG) was recorded using 21 channels (one position over the zygomatic arch) according to the International 10–20 system and digitized with a sampling frequency of 256 Hz (Refa8‐32, TMS International). Simultaneous digital video was recorded (1920 × 1080 pixels) with a camera on the EEG setup (Axis P1375 Network Camera, Axis Communications). Eye movements were recorded using a Smart Eye Pro system (Smart Eye) sampling at 60 Hz. The system used binocular tracking with infrared illumination and allowed moderate head movement. Calibration was performed before each drive, and calibration quality was verified before data collection.

#### 2.2.3 Car simulator

The static car simulator consisted of a driver seat, steering wheel, pedals, eye tracking system, display for the driving environment and speedometer, a computer, and four cameras that captured the driver's face, body, and feet.

### 2.3 Analysis

EEG and video were reviewed by a neurophysiologist (J.S.). The simulator recordings were reviewed by Volvo engineers (E.N., M.Z.Z.). For Subject 1, the eye tracking recording failed (i.e., no data recording). For Subject 3, the eye tracker failed to get a reliable tracking due to the subject having very narrow eye openings while driving. Because it was necessary to make manual annotations from the video recordings for two of three subjects, we chose to do so for all three for consistency. Gaze direction, eye blinks, and saccades were manually annotated by E.N. from the video recordings. The frequency of eye blinks and saccades, and the eyes‐on‐road during the seizures, were compared to 4–5‐min baseline epochs preceding each seizure.

### 2.4 Ethical approval

The Swedish Ethical Review Authority approved the study (2023–6060).

## 3 RESULTS

We included 10 participants (Table 1); all had high seizure frequencies and a history of focal impaired awareness seizures. Only one had held a driver license.

**TABLE 1:** Study cohort, demographics, and epilepsy characteristics.

Characteristic	Value
Age, years, median (range)	32 (22–59)
Number of seizures in past 2 months, median (range)	40 (6–200)
Current number of antiseizure medications, median (range)	3 (1–4)
Female sex, *n* (%)	9 (90%)
Epilepsy etiology, *n* (%)	
Unknown	5 (50%)
MCD	4 (40%)
Immune‐mediated	1 (10%)
Semiology reported before driving, *n* (%)	
Focal aware seizures	4 (40%)
Focal impaired awareness seizures	9 (90%)
Ever bilateral tonic–clonic seizures	5 (50%)
Drug‐resistant epilepsy	9 (90%)
EEG‐verified seizure previously	10 (100%)

Abbreviations: EEG, electroencephalography; MCD, malformation of cortical development.

During 10 recording days (one subject/day), we recorded three definite seizures and one possible electrographic seizure in three subjects. Participants found maneuvering difficult and mistakes were common also without seizures.

### 3.1 Subject 1 Seizure 1

#### 3.1.1 Clinical observations and EEG

There were no apparent symptoms. The subject kept driving. On EEG, there was sudden onset of high‐frequency activity with low amplitude in P8 and T8, then progression of activity/evolving pattern with increased amplitude and lowered frequency and spread to Zyg2, F8, P4, and O2. There was a sudden end. Duration was 12 s. It resembled previously recorded seizures.

#### 3.1.2 Driving and eye movements

The subject drove without obvious change in performance. The eye blink and saccade frequencies were slightly lower during the seizure compared to baseline, whereas the eyes‐on‐road percentage did not differ (Table 2).

**TABLE 2:** Eye behaviors during BL and seizure segments.

	Dur, s	Blink freq	Saccade freq	On‐road, %
Subject 1				
BL	60	12	45	96.8
BL	60	12	56	97.4
BL	60	11	43	93.8
BL	60	17	45	92.6
Sz 1	14.0	8.6	21.4	96.7
BL	60	10	69	99.6
BL	60	15	47	96.7
BL	60	9	57	91.4
BL	60	14	66	90.9
Sz 2	16	22.5	48.8	90.4
Subject 2				
BL	60	11	18	96.0
BL	60	10	32	92.1
BL	60	19	37	86.2
BL	60	15	27	92.3
BL	60	22	29	92.0
Sz 1	12.0	10.0	30.0	66.1
Subject 3				
BL	60	14	26.6	100
BL	60	11	15.0	99.4
BL	60	19	19.1	100
BL	60	23	23.9	98.4
BL	60	22	3.8	100
Sz 1	6	30	40	100

*Note*: On‐road indicates gaze direction.

Abbreviations: %, percentage of time; BL, baseline; Dur, duration; freq, number per minute; Sz, seizure.

### 3.2 Seizure 2

#### 3.2.1 Clinical observations and EEG

There were no visible clinical symptoms. The subject reported obscured vision and loss of focus followed by confusion and nausea after the seizure. Immediately after the electrographic seizure, the patient let go of the steering wheel, stopped, and called for attention. EEG showed seizure activity identical to that described above. Duration was approximately 11 s.

#### 3.2.2 Driving and eye movements

The subject drove as in baseline until driving to the side of the road and stopping the car. The eye blink frequency was slightly higher during the seizure, whereas the saccade frequency and eyes‐on‐road percentage did not differ from baseline (see Table 2).

### 3.3 Subject 2

#### 3.3.1 Clinical observations and EEG

Approximately 4.5 s after start of electrographic seizure activity, subject let go of the steering wheel and did not want to drive anymore, reporting being tired. It was not obviously different from the ending of previous driving sessions. On EEG, seizure activity started with solitary spike‐and‐slow‐wave in F3/F7/C3/T7/P7/Zyg1/P3 followed by a suppression of activity and subsequent fast sharp activity in C3/F3/Fp1, after a few seconds also evolving rhythmic slow activity in T7/F7/P7/C3/P3/Zyg1 with sudden end. Duration was approximately 12 s. It resembled previously recorded seizures.

#### 3.3.2 Driving and eye movements

Subject drove as in baseline until driving to the side of the road and stopping the car. Saccades and eye blink frequencies were not different from baseline, whereas the eyes‐on‐road percentage was lower (Table 2) due to more time spent looking toward the speedometer.

### 3.4 Subject 3

#### 3.4.1 Clinical observations and EEG

There were no apparent clinical symptoms. Afterward, the subject reported feeling of seizure onset. On EEG, there were short episodes with rhythmical low‐frequency activity in Cz intermixed with spike potentials (unfortunately poor signal in adjacent C4; previous EEGs had subclinical seizures in Cz/C4). Duration of episodes was 1–6 s. The longest episodes also had a hint of progression/evolving pattern and were considered possible seizures or brief potentially ictal rhythmic discharges. It resembled previously recorded subclinical seizures, but the patient had several previous seizure types with different onset.

#### 3.4.2 Driving and eye movements

The subject drove as in baseline. Saccade and eye blink frequencies were higher compared to baseline, whereas the eyes‐on‐road percentage did not change (Table 2).

## 4 DISCUSSION

In our pilot study, none of the registered seizures caused consistent changes in driving behavior, and there were only small and inconsistent changes in eye blink and saccade frequencies. It was not possible to distinguish these episodes from baseline driving using the vehicle sensors.

Our investigation holds several lessons for future investigations. For safety reasons, the study was performed in an epilepsy monitoring unit (EMU). No tonic–clonic seizures occurred, but the setup was perceived as safe, and large‐scale recordings seem possible. However, despite 10 experimental days with subjects with high seizure frequency, we were still only able to record four brief seizures of questionable relevance for driving. Future studies need to be scaled for extensive data collection and could perhaps involve medication withdrawal in conjunction with surgical evaluations.

Participants should receive more training or hold a valid driver license to ensure reliable evaluation of driving. Errors in distancing and positioning were frequent during baseline conditions. The simulator could perhaps be more realistic; static simulators cannot reproduce any lateral or longitudinal accelerations or yaw, pitch, or roll sensations. This leads to unnatural motion perception and a less realistic driving experience, as driving relies on visual cues only.

It is unclear whether the seizures did result in reduced driving ability. The recorded seizures were not observed by experiment staff but only by the clinical neurophysiologist reviewing the EEG and in some cases by the subject themselves. Subtle seizures can affect driving ability, but mostly if consciousness is affected.^13^, ^14^ The baseline variability in driving performance in our subjects could be another contributor to our negative findings. Notably, two of three participants were able to recognize the onset of a seizure while driving, promptly maneuver their vehicles to the side of the road, and cease to drive. This raises interesting questions about integrating driver‐initiated signals into vehicle safety systems.

From our very limited data, it does appear challenging for eye tracking to detect brief focal seizures that lack prominent motor symptoms. This is not surprising. Eye tracking is primarily used to monitor signs of sleepiness and distraction, typically by measuring parameters like duration of eye closure or gaze deviation from the road ahead. We found no consistent alterations in eye blinking, saccades, or gaze directions in our subjects during the brief seizures captured in our study, but some subjects did deviate (up or down) from their preictal baseline in blink frequency or saccades. Larger datasets are needed in terms of both number and type of seizures. More advanced data analysis techniques, including machine learning, should be included in subsequent analyses, and might be able to identify trends in eye movements not detectable with our manual approach.

The main conclusion is that collecting sufficient and relevant data will be difficult. We recorded only four brief seizures that were not very helpful in assessing the capacity of eye movements or driving behavior. This translates into a need for many hundreds of sessions to build datasets of sufficient size for machine learning applications. Common data elements and a collaborative—perhaps global—approach seem necessary. It should also be explored whether data can be alternatively collected; blinking and saccade data for training of algorithms can perhaps be obtained from EMU videos, but studying gaze requires more carlike settings.

Detecting subtle seizures in car drivers is a challenging task. There is a great need for technologies and protocols that can detect subtle changes in neurological or behavioral states indicative of seizure activity, even when overt physical symptoms are absent. Given the loss of independence associated with lost driving privileges,^2^ research into technology allowing persons with epilepsy to operate vehicles safely should remain a priority.

## AUTHOR CONTRIBUTIONS

*Concept and design:* Emma Nilsson, Mirta Zelenika Zeba, Joakim Strandberg, Johan Zelano, and Peter Lundgren. *Acquisition, analysis, and interpretation:* Mirta Zelenika Zeba, Emma Nilsson, Joakim Strandberg, and Johan Zelano. *Drafting of manuscript:* Johan Zelano. *Critical revision of manuscript:* all authors.

## FUNDING INFORMATION

The study was funded by Volvo Cars and the Swedish State through the ALF agreement.

## CONFLICT OF INTEREST STATEMENT

J.Z. reports (outside the submitted work) speaker/advisory board honoraria from Eisai, UCB, Orion Pharma, Sanofi, and Angelini Pharma. As an employee of Sahlgrenska University Hospital, J.Z. served as an investigator in clinical trials for UCB, Bial, SK Life Science, Angelini Pharma, and GW Pharma (no personal compensation). P.L. reports serving in a medical advisory role for Volvo Cars. None of the other authors has any conflict of interest to disclose. We confirm that we have read the Journal's position on issues involved in ethical publication and affirm that this report is consistent with those guidelines.

## Data Availability

The data that support the findings of this study are available on request from the corresponding author. The data are not publicly available due to privacy or ethical restrictions.
